# Risk of suicide during pregnacy and postpartum period

**DOI:** 10.1192/j.eurpsy.2022.2214

**Published:** 2022-09-01

**Authors:** A. Duarte, M. Ribeiro, J. Lopes, S. Oliveira, P. Martins

**Affiliations:** 1 Centro Hospitalar Lisboa Norte - Hospital Santa Maria, Psychiatry And Mental Health, Lisboa, Portugal; 2 Centro Hospitalar Lisboa Norte, Psychiatry, Lisboa, Portugal; 3 USF Ermesinde, Usf Ermesinde, Ermesinde, Portugal

**Keywords:** Postpartum, Suicide, Pregnancy

## Abstract

**Introduction:**

Pregnancy and the postpartum are generally characterized by positive feelings and expectations but they may also disguise maternal stress and difficulties. These are typical periods for the onset or relapse of psychiatric symptoms and disorders. Even though suicide during pregnancy and postpartum is rare, it is among the leading causes of maternal perinatal mortality.

**Objectives:**

To provide an overview on the risk of suicide during pregnancy and postpartum.

**Methods:**

PubMed database was searched using combinations of the terms “suicide”, combined with “pregnancy” and “depression”.

**Results:**

The major risk factors for suicidal ideation are previous suicide attempts, self-harm, current or past history of psychiatric disorder, young maternal age, being unmarried, an unplanned pregnancy, substance use disorders, lack effective psychosocial support and discontinuation of psychotropic drugs. Pregnant women with suicidality behavior have also an increased risk for various adverse obstetric outcomes, including miscarriage, preterm delivery, maternal hemorrhage, and stillbirth. Furthermore, the postpartum period is often associated with the onset of mood and psychotic disorders with an increased risk of both suicide and infanticide. Women who have suffered from serious psychiatric conditions either after childbirth or in other phases of life should be informed about the possibility of relapse after subsequent pregnancies, thus presenting a higher risk of suicide.

**Conclusions:**

During pregnancy and postpartum, it is fundamental to investigate suicide risk, including suicidal ideation, thoughts, and intent, especially (but not only) in women affected by mental pathology. Moreover, maternal suicide behaviour affects the child’s neuropsychological development and can also increase the infant´s suicide risk.

**Disclosure:**

No significant relationships.

